# Congenitally Malformed Hearts: Aspects of Teaching and Research Involving Medical Students

**DOI:** 10.3390/jcdd8040034

**Published:** 2021-03-28

**Authors:** Catherine C. Pickin, James Castle, Vibha Shaji, Adeolu Banjoko, Aimee-Louise Chambault, Anna N. Seale, Anthony Lander, Chetan Mehta, Adrian Crucean

**Affiliations:** 1 Department of Anatomy, College of Medical and Dental Sciences, University of Birmingham, Birmingham B15 2GW, UK; catherine.pickin@nhs.net (C.C.P.); james.castle@nhs.net (J.C.); vibha.shaji@nhs.net (V.S.); adeolu.banjoko@nhs.net (A.B.); aimee-louise.chambault@nhs.net (A.-L.C.); 2 Department of Paediatric Cardiology, Birmingham Women’s and Children’s Hospital, Birmingham B4 6NH, UK; annaseale@nhs.net (A.N.S.); chetan.mehta1@nhs.net (C.M.); 3 Institute of Cardiovascular Sciences, University of Birmingham, Birmingham B15 2TT, UK; 4 Department of Paediatric Surgery, Birmingham Women’s and Children’s Hospital, Birmingham B4 6NH, UK; t.lander@nhs.net; 5 Department of Paediatric Cardiac Surgery, Birmingham Women’s and Children’s Hospital, Birmingham B4 6NH, UK

**Keywords:** congenital heart defects, congenitally corrected transposition of the great arteries, right isomerism of the atrial appendages, left isomerism of the atrial appendages, right aortic arch, double aortic arch, abdominal heterotaxy

## Abstract

To appreciate congenital heart disease fully, a detailed understanding of the anatomical presentation, as well as the physiology, is required. This is often introduced at an advanced stage of training. Professor Anderson has been influential in the Clinical Anatomy Intercalated BSc programme at the University of Birmingham, in particular in his teaching on Sequential Segmental Analysis. This article describes the experiences of the latest cohort of students on this programme, who undertook varying research projects using the Birmingham Cardiac Archive, with the guidance of Professor Anderson. The projects outlined include various aspects of isomerism, encompassing both the cardiac and abdominal manifestations, as well as details of congenitally corrected transposition of the great arteries and prenatally diagnosed right aortic arch and double arch. These studies all aimed to increase the knowledge base of their respective cardiac malformations and provide a basis for further research.

## 1. Introduction

The study of congenital heart disease was considered to require a substantial layer of anatomy and physiology enriched with a solid knowledge of clinical cardiology and multiple elements of general surgery, cardiac surgery and intensive care. Hence, it is still the case that most medical professionals start this advanced specialisation once they have completed their training in areas such as paediatrics, cardio-thoracic surgery and cardiology. It may seem counterintuitive to propose such complex topics to medical students still early in their careers.

We would like to share here the experience with our latest cohort of year 3 and 4 students in BSc Intercalation in Clinical Anatomy at the University of Birmingham who completed their degree projects in congenital heart disease. This brings to light several important roles that Professor Robert H. Anderson generously undertook in the past 8 years in relation to the Birmingham Cardiac Archive.

Professor Anderson while allegedly retired, visited the Birmingham Children’s Hospital on numerous occasions since 2013 in order to classify a significant and vast archive of more than 2000 preserved malformed hearts. He painstakingly contributed to catalogue the specimens based on the updated nomenclature, delivered numerous high quality teaching sessions, participated in numerous clinical discussions of complex patients, mentored the current curator of the archive, and advised for a robust governance process for the archive committee including a novel digital database for the systematic documentation of the hearts examined. He also contributed to initiate the Intercalation Programme with the University of Birmingham, an optional one-year programme taken between third and fourth or fourth and fifth years during the MBChB programme. Professor Anderson understood very early on that a dedicated clinical and academic team would be able to overcome any obstacles raised against achieving excellent results with such a programme. He has taught countless students on the programme the intricacies of Sequential Segmental Analysis to properly understand the anatomy of congenitally malformed hearts. The University of Birmingham is now running the 5th such programme and the results have been nothing short of very satisfactory: all students achieved first class honours following an external examination at the end of the intercalation year, some students were able to publish in high quality journals in our specialty and presented to highly ranked international conferences such as EACTS (European Association of Cardio-Thoracic Surgery) meeting or ESC (European Society of Cardiology) Development meeting, and some students became enthused with the paediatric cardiac specialty and are now following it as a medical career. Nevertheless, the most important achievement was to witness their transformation during the academic year: their unleashed curiosity blended with hard work and youthful passion which eroded any barriers to understanding even of the most complex aspects of cardiac anatomy in malformed hearts.

This is just another aspect of the multiple pronged legacies that Professor Anderson created during his considerably long career as a pioneer in a domain that will continue to marvel and attract generations of healthcare professionals, academic researchers and teachers in the decades to come. To his credit we describe a few projects collated from the latest academic year in a student format. In these projects, hearts with atrial appendage isomerism and congenitally corrected transposition of the great arteries (ccTGA) were identified in the Birmingham Cardiac Archive. The complex cardiac morphology was analysed and documented according to Sequential Segmental Analysis. Care has been taken to present only partial results as the separate detailed papers will contain the complete data for each separate project. We are thankful to the students and their supervisors who agreed to share their work for this special article.

## 2. Left Isomerism of the Atrial Appendages–Catherine Pickin

Atrial appendage isomerism is a rare form of congenital heart disease characterised by mirror imaged appendages of the heart [1]. It is associated with a heterogenous spectrum of cardiac morphology, extracardiac associations, presentation, management and outcomes [1,2,3,4].

This heterogeneity has led to controversy over the diagnosis and classification of this condition, which has also been known as ‘heterotaxy’ and ‘bodily isomerism’, particularly with regard to its left and right subtypes [5,6]. Professor Anderson and colleagues have consistently shown in post-mortem morphological studies that hearts with atrial appendage isomerism can and should be classified according to their most constant morphological component: the extent of the pectinate muscles within the appendages of the heart [1,7]. Left atrial appendage isomerism is defined by the presence of bilateral morphologic left atrial appendages, themselves defined by the pectinate muscle being confined to the appendage, not extending to the crux of the heart [1].

**Figure 1:**
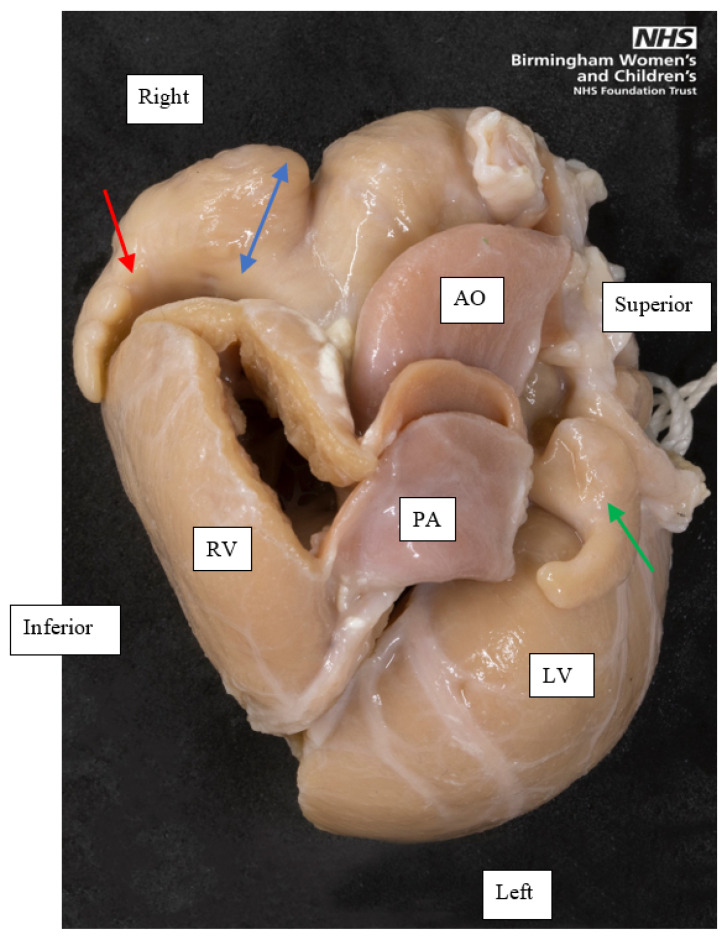
A superior view of the base of a heart with left atrial appendage isomerism, showing the external appearance of both appendages. The left-sided appendage, indicated by the green single-headed arrow, is small and tubular. The right-sided appendage, indicated by the red single-headed arrow, is tubular at its distal parts, but its proximal part is broad based, shown by the blue double-headed arrow. The pectinate muscle of both appendages was confined to the appendage (not shown in this image), confirming this as a heart with left atrial appendage isomerism. Orientation points displayed in an attitudinally correct fashion. AO, Aorta; PA, Pulmonary artery; RV, Right ventricle; LV, Left ventricle.

**Figure 2:**
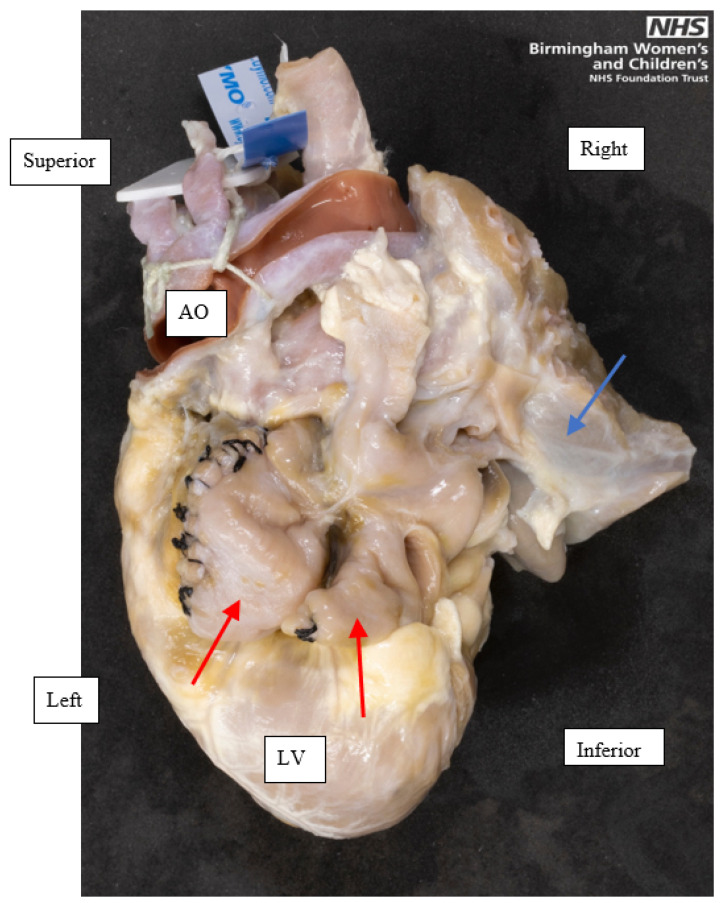
A left posterior view of a heart with left atrial isomerism, showing the juxtaposed morphologic left atrial appendages, indicated by the red single-headed arrows. Both appendages are tubular and had a narrow base to the atrium. Parts of the lungs, indicated by the blue single-headed arrow, have been moved to the right and superiorly to expose the appendages. This heart had been operated on in life, indicated by the black stitches in both appendages. Orientation points displayed in an attitudinally correct fashion. AO, Aorta; LV, Left ventricle.

Seven hearts with left atrial appendage isomerism were analysed. We particularly noted the variation in external shape of the appendages of the same heart, when the extent of the pectinate muscle in both indicated left atrial appendage isomerism (Figure 1). One heart had juxtaposed appendages, meaning assessment of the pectinate muscle was difficult (Figure 2). These highlighted the difficulty in assessing the morphological diagnosis of hearts with atrial appendage isomerism.

**Figure 3:**
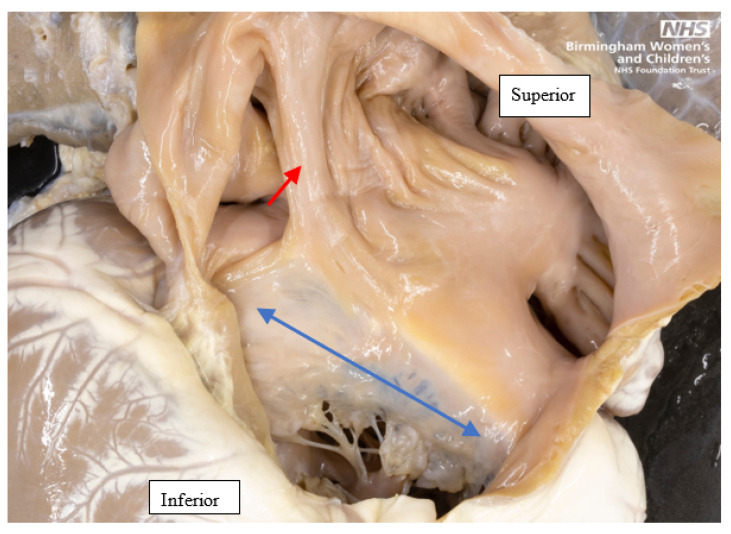
Antero-superior view of the internal aspect of the common atrium and the common AV valve of a heart with left atrial appendage isomerism. The anterior wall of the atrium has been dissected and reflected superiorly and laterally. A septal strand on the posterior wall of the atrium is shown by the red single-headed arrow. The common AV valve, within the common AV junction, is shown by the double headed blue arrow.

We found atrial septum anomalies (Figure 3) in all seven hearts, consistent with the landmark study by Professor Anderson and colleagues in which they found the atrial septum was rarely properly formed in left atrial appendage isomerism [1]. We also found incidences of common atrioventricular (AV) valve (5/7, 71%) and common AV junction (6/7, 86%) similar to those found by Anderson [1], indicating the importance of using consistent diagnostic criteria so studies are able to be accurately compared.

This research, instigated by Professor Anderson, continues the much needed exploration into the cardiac morphology and associations in the heterogeneous condition of left atrial appendage isomerism.

In parallel we also conducted a large 20-year retrospective cohort study of cardiac morphology and clinical outcomes in right and left atrial appendage isomerism at Birmingham Women’s and Children’s NHS Foundation Trust (BWCH). We found 138 patients with atrial appendage isomerism of whom 59% (81/138) had left atrial appendage isomerism.

## 3. Right Isomerism of the Atrial Appendages–James Castle

Right atrial appendage isomerism is defined by the presence of bilateral morphologic right atrial appendages, themselves defined by pectinate muscle extending around the atrioventricular junction.

21 hearts with right atrial appendage isomerism were identified. In addition, a descriptive study examining the patient factors, treatments and outcomes of patients with RAI treated by the Cardiology and Cardiac Surgery departments at BWCH was carried out. Through automated key term searches followed by manual validation, the retrospective analysis of the cardiology departmental records identified 57 patients with RAI.

The greatest utility for the archive with respect to cardiac morphology is the unsurpassed detail with which specimens can be examined compared with in vivo imaging techniques. It has been shown in the literature that although extremely accurate, modern imaging systems are not perfect, having particular issues with valvular morphology and anomalous venous connections [8]. Given that these features are common in hearts with RAI, the importance of physical specimens is perhaps even greater than for simpler cardiac malformations. In addition, working with a physical specimen allows a greater understanding of the three-dimensional relationship of features within the malformed heart which cannot be fully appreciated with two-dimensional imaging or even computerised reconstructions.

## 4. Abdominal Heterotaxy and Cardiac Isomerism–Vibha Shaji

Isomerism of the atrial appendages (AAI) only describes isomerism within the heart. However, it is associated with numerous extracardiac manifestations encompassing malformations of the stomach, spleen, liver, gallbladder, pancreas and intestines; this is acknowledged by the currently prevalent terms ‘heterotaxy’, ‘heterotaxy syndrome’ and ‘atrial isomerism’, which are used as synonyms for situs ambiguous, asplenia or polysplenia syndromes. When discussing the abnormalities of situs in the abdomen, ‘heterotaxy syndrome’ was deemed appropriate as the term atrial isomerism cannot apply in the way it is applied to the heart since paired structures are being discussed [9]. Moreover, the abdomen demonstrates inconsistent features in comparison to the isomeric atrial appendages.

Sequential segmental analysis enables a thorough analysis of cardiac morphology to understand the patient’s condition better. There is often agreement that any abdominal surgery should be undertaken after cardiac palliation [10] due to the higher underlying risk of mortality associated with particular cardiac lesions. For this reason, it has made sense in the past to focus on developing a descriptive manner to characterise cardiac morphology. Recently, however, given that patients are surviving for long enough to exhibit signs of abdominal abnormalities [11], it seems rational to foster a similar method of analysis of the abdomen to allow for better guided patient care.

To this end, the literature was reviewed and showed that most studies address the need for exploring the arrangement of abdominal organs in greater detail, with some even stating that they will give the “precise arrangement” of these viscera [12]. However, these very papers frequently provide no more detail than the organ laterality [13,14]. Moreover, while most document the side of these organs, as this can be beneficial to a surgical approach, very few relate these to their clinical significance [15,16,17].

Therefore, a retrospective observational study of all patients with AAI who had malrotation was designed at BWCH using more imaging modalities than all other previous studies to allow for a more profound analysis of the anatomy of the 23 patients included. A systematic approach to describing the abdominal viscera was also created, the details of which are to be described in a future manuscript.

All patients included within the study displayed unique anatomical morphology, emphasising the importance of appropriate description to guide clinical management. However, none had a complete anatomical description available. Given the clinical implications of these varied presentations, this should be obtained as a standardised aspect of management, especially given the increased life span of these patients [11]. The newly proposed systematic approach would facilitate this, providing several key additional benefits. These include the standardisation of reporting of the morphology present and complete visualisation and description of the viscera, ensuring that all relevant anatomical information is collated.

## 5. Prenatally Diagnosed Right Aortic Arch and Double Arch: Incidence, Associations and Outcomes-Aimee-Louise Chambault

Right aortic arch (RAA) and double aortic arch (DAA) abnormalities can form structures called vascular rings (VR) [18]. VR can encircle the trachea and oesophagus and cause compression, which consequently produces symptoms [19]. There is limited literature regarding the incidence, associations and outcomes of both RAA and DAA abnormalities in a prenatally diagnosed cohort. Therefore, this subject was explored further as part of this dissertation project, via a retrospective observational study conducted at BWCH. This was in addition to the production of a treatment pathway for these patients within this institution. This study provided pilot data for a nationwide collaborative study on this lesion.

This project reviewed patients who received a prenatal diagnosis of a RAA or DAA following referral to BWCH. The primary inclusion criterion for this project was that patients must have had a prenatal diagnosis of a RAA/DAA. Exclusion criteria for this project included cases that were only postnatally diagnosed with a RAA/DAA. Additionally, patients with a prenatal diagnosis of concurrent major congenital heart disease were also excluded. The final cohort of patients included 160 in the prenatal cohort and 123 patients in the postnatal cohort. Some of the key outcomes explored included genetic testing results, additional cardiac/extracardiac abnormalities, as well as surgical outcomes for those patients who underwent surgery.

Results of this project found that the incidence of an isolated RAA and DAA was 9.1 and 1.3 per 10,000 women undergoing fetal second trimester anomaly screening, respectively. Since the introduction of the three-vessel and trachea view into the second trimester fetal anomaly scan in 2015, the BWCH fetal medicine department was referred approximately 45 cases per annum of isolated RAA/DAA; previously this had been less than 3. Postnatally, 14 patients had genetic abnormalities, 19.2% (14/73), of 73 patients with known genetic test results. Extracardiac abnormalities were found in 13.0% (16/123) of patients in the postnatal cohort. In total, 17.1% (21/123) of patients underwent surgery, and the anatomy of these patients was either a DAA, 52.4% (11/21) or a RAA with an aberrant left subclavian artery, 47.6% (10/21).

For this project, it was important to understand the embryological origins of the aortic arch, and the subsequent formation of RAA and DAA abnormalities. This was achieved using articles and books authored by Professor Anderson, as well as the embryology lectures that he delivered as part of the wider clinical anatomy course [18,20,21]. These helped shed light on a relatively complicated developmental lesion, as well as aid in understanding of some of the more complex anatomical presentations of RAA abnormalities in particular.

In conclusion, the results of this project were able to establish the incidence, associations and outcomes of patients with a prenatally diagnosed RAA/DAA referred to BWCH for diagnosis. These preliminary results have provided valuable data at a local level, and data collected will also be utilised in a nationwide project on the same subject matter. All of these factors will help to better establish a way forward, particularly in terms of management, for patients with this anatomical abnormality.

## 6. Congenitally Corrected Transposition–Adeolu Banjoko

Congenitally corrected transposition of the great arteries (ccTGA) is a rare congenital cardiac malformation impacting 0.5% of those born with congenital heart disease [22]. ccTGA has both atrioventricular and ventriculoarterial discordant connections, as described by Sequential Segmental Analysis [23,24]. There are four key malformations that are commonly associated with ccTGA. These include a ventricular septal defect, obstruction of the right ventricular outflow tract, Ebsteinoid’s malformation of the tricuspid valve, and morphological right ventricular (mRV) hypoplasia [18]. While ccTGA is coined a “corrected transposition” due to having a relatively normal circulation, these associated malformations often have immediate impact on function and survival in ccTGA patients [18].

This project aimed to describe the associations between prenatal and postnatal morphology and clinical outcomes in a cohort of prenatally diagnosed ccTGA patients at BWCH. An analysis of 22 archived ccTGA hearts was used to supplement clinical images and descriptions, to better understand the morphology of these patients.

The 22 specimens in the BWCH archive were analysed as part of this project. The examination protocol was according to the blood flow, with variables collected to support the clinical data and build upon the current morphological knowledge of ccTGA in the literature. One innovative inclusion in our examination protocol was the assessment of ventricular septal defect (VSD) topography from the mRV. This is in continuation from a recent publication by Arribard et al. [25], in which they identify the mRV septum of ccTGA to be an identical mirror image of that in normal hearts. Accordingly, VSD assessment should be standardised to the mRV because this allows for the use of the criteria widely agreed in isolated VSD. When capturing these VSD, specialised mirrors were used to make the mRV appear right sided.

**Figure 4:**
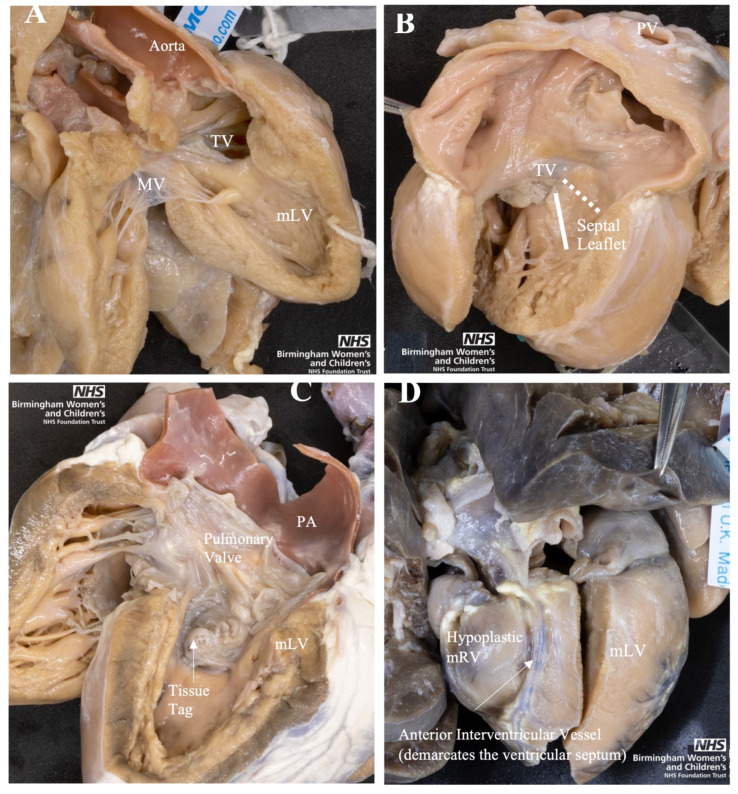
Four Associated Anomalies of ccTGA. (**A**) Peri-membranous VSD with aorta overriding and tricuspid valve straddling, from the morphological left ventricle (mLV). (**B**) Septal leaflet is plastered to septum of morphological right ventricle (mRV). Dashed line shows normal atrioventricular junction and solid line shows apical displacement of septal leaflet. (**C**) Subpulmonary obstruction due to tissue tag of septal leaflet of tricuspid valve. (**D**) Ventricular hypoplasia of the mRV. MV, Mitral Valve; PV, Pulmonary Vein; TV, Tricuspid Valve; mLV, morphological left ventricle; mRV, morphological right ventricle; mLA: morphological left atrium.

All four associated anomalies were represented in the archive cohort (Figure 4). Of particular interest was capturing the unique “side by side” ventricular positioning of the ventricles in ccTGA. The clinical data found mRV hypoplasia was overly suspected prenatally. A contributor to this clinical finding could be the unusual positioning of the ventricles in ccTGA. When assessed in the limited echocardiographic cross sections available prenatally, this unique positioning of the ventricles can result in underestimation of the size of the mRV [26]. An awareness of this morphology, provided by this analysis in the archive, can help clinicians improve their fetal morphological assessment in ccTGA patients.

**Figure 5:**
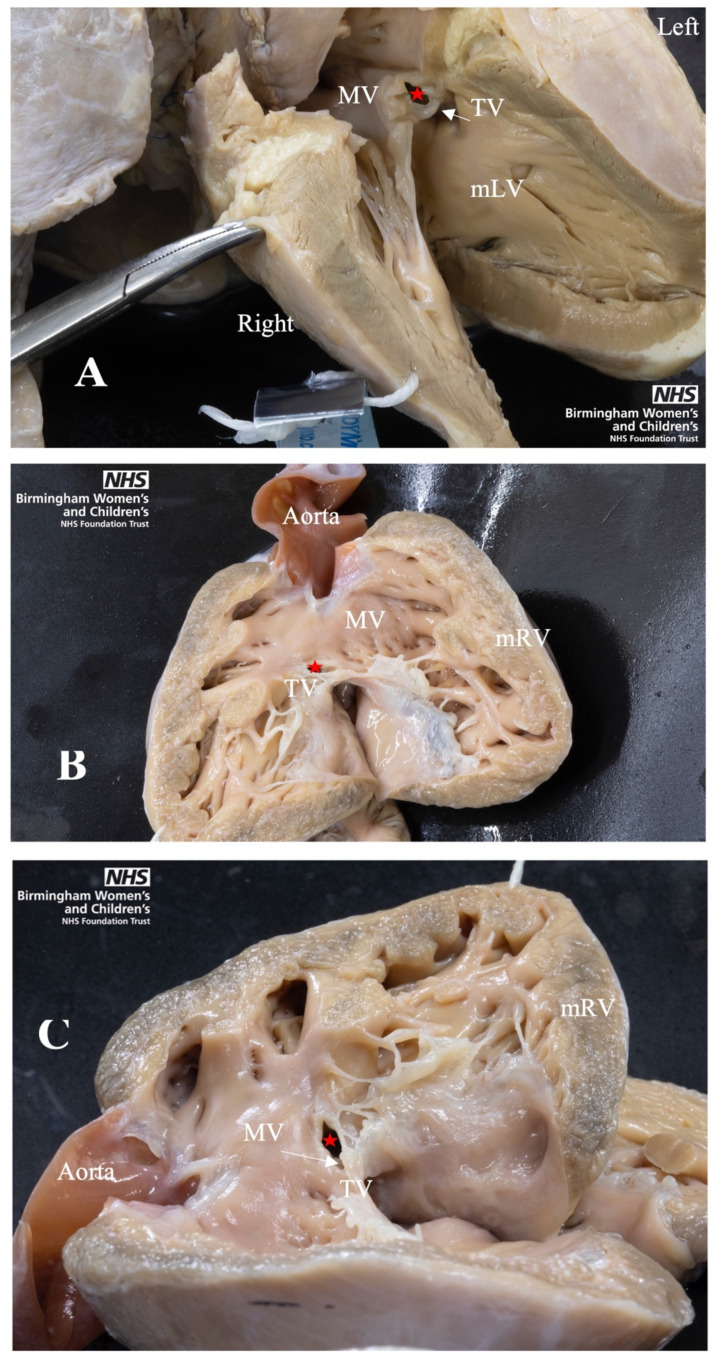
Four Associated Anomalies of ccTGA. (**A**) Photo from mLV showing Peri-membranous VSD. (**B**) Photo from mRV showing Peri-membranous VSD. (**C**) Photo from mRV using mirror so the ventricle appears right sided. Geographies of VSD assessed as central. Star: VSD; MV, Mitral Valve; TV, Tricuspid Valve; mLV, morphological left ventricle; mRV, morphological right ventricle.

Furthermore, all the ccTGA hearts with VSD had their topography assessed from the mRV. Three of these hearts had their VSD photographed from the mRV using a mirror, to represent the VSD appearing right-sided, similar to concordant atrioventricular connections (Figure 5). We found the VSD in the archive has more diverse topographies when comparing to Arribard et al. [25]. While the majority of their ccTGA hearts had an outlet perimembranous VSD upon assessment from the mRV, our archive had a mix of 2 central, 6 inlet, 1 inlet to outlet, and 4 outlet perimembranous VSD [25].

This project described the morphology of both clinical patients and archive specimens with ccTGA. These specimens also contribute to current literature by assessing the VSD from the mRV, using a mirror to appear right sided. This exploration of the morphology helps improve understanding of the morphological spectrum of ccTGA patients.

## 7. Conclusions

This article attempted to provide an example of the extensive depth and breadth of Professor Anderson’s contribution to the field of congenital heart disease. In an age of continuous super-specialisation, a polymath-like figure like him has been able not only to cross boundaries in terms of various clinical specialties but also to unite different levels of expertise such as students and beginners in the medical field, with world-renowned developmental or imaging scientists, archive curators and basic science researchers. Professor Anderson continues to inspire and guide new generations of young medical students in the quest for a better understanding of congenital heart disease.

## Data Availability

The data presented in this study are available on request from the corresponding author. The data are not publicly available due to separate submissions being prepared for individual papers and also data being part of multicentre studies.
